# Bioethanol and lipid production from the enzymatic hydrolysate of wheat straw after furfural extraction

**DOI:** 10.1007/s00253-018-9081-7

**Published:** 2018-05-26

**Authors:** Jule Brandenburg, Ieva Poppele, Johanna Blomqvist, Maris Puke, Jana Pickova, Mats Sandgren, Alexander Rapoport, Nikolajs Vedernikovs, Volkmar Passoth

**Affiliations:** 1 Department of Molecular Sciences, Swedish University of Agricultural Sciences, P.O.-Box 7015, S-75007 Uppsala, Sweden; 2 Institute of Microbiology and Biotechnology, University of Latvia, Jelgavas Str., 1-537, Riga, LV-1004 Latvia; 3 Present Address: Faculty of Science and Technology, Norwegian University of Life Sciences, Ås, Norway; 4 Latvian State Institute of Wood Chemistry, Dzerbenes Str. 27/345, Riga, LV-1006 Latvia

**Keywords:** Wheat straw, Lignocellulose, Furfural production, Ethanol, Biodiesel

## Abstract

This study investigates biofuel production from wheat straw hydrolysate, from which furfural was extracted using a patented method developed at the Latvian State Institute of Wood Chemistry. The solid remainder after furfural extraction, corresponding to 67.6% of the wheat straw dry matter, contained 69.9% cellulose of which 4% was decomposed during the furfural extraction and 26.3% lignin. Enzymatic hydrolysis released 44% of the glucose monomers in the cellulose. The resulting hydrolysate contained mainly glucose and very little amount of acetic acid. Xylose was not detectable. Consequently, the undiluted hydrolysate did not inhibit growth of yeast strains belonging to *Saccharomyces cerevisiae*, *Lipomyces starkeyi*, and *Rhodotorula babjevae*. In the fermentations, average final ethanol concentrations of 23.85 g/l were obtained, corresponding to a yield of 0.53 g ethanol per g released glucose. *L. starkeyi* generated lipids with a rate of 0.08 g/h and a yield of 0.09 g per g consumed glucose. *R. babjevae* produced lipids with a rate of 0.18 g/h and a yield of 0.17 per g consumed glucose. In both yeasts, desaturation increased during cultivation. Remarkably, the *R. babjevae* strain used in this study produced considerable amounts of heptadecenoic, α,- and γ-linolenic acid.

## Introduction

Lignocellulose is very attractive as raw material for biofuel and chemicals’ production, as it is the most abundant biomass on Earth and has less impact on feed and food production and results in greater greenhouse gas savings compared to food grade, first generation substrates (Gnansounou 2010, Özdenkçi et al. 2017). However, due to its recalcitrance, energy intense and expensive thermo-chemical and enzymatic pretreatments are required to decompose lignocellulose into its basic building blocks, to make them available for further processing (Özdenkçi et al. 2017). During pretreatment, a variety of compounds such as furfural and acetic acid are formed that may act as inhibitors in subsequent fermentation processes (Jönsson and Martin 2016). On the other hand, furfural has been identified as one of the major biomass-based platform chemicals for chemical industry to produce biofuels, fuel additives, and other high-value compounds. This natural dehydration product of xylose and other pentoses is industrially produced from hemicellulose-rich substrates such as corncobs or sugarcane bagasse.

The global annual production of furfural at 2010 has been reported to be around 300,000 t (Dashtban et al. 2014), and it is expected that at 2020, the global furfural market will reach 650,000 t (GrandViewResearch 2015). Currently, no technology for chemical synthesis of furfural does exist, and thus, furfural is the most abundant industrial chemical that is derived from lignocellulose. However, its global market volume is largely stagnating. This is mainly due to the inefficient processes of current furfural production, which makes it difficult to compete with fossil-based chemicals. The majority of current industrial furfural processes are based on a technology that was developed in the 1920’s by Quaker Oats (Iowa, USA). This process does not reach 50% of the potential maximum yield. Apart from this, current industrial furfural production processes tend to also damage the cellulose fraction of the biomass, making it very difficult to convert this polysaccharide to glucose and further to ethanol (Win 2005, Lange et al. 2012, Cai et al. 2014, Rapoport et al. 2014, Machado et al. 2016).

At the Latvian State Institute of Wood Chemistry, novel methods of thermochemical treatment of lignocellulose have been developed, enabling a higher furfural yield compared to previously established methods. This process is based on the injection of a strong acid and certain salts, enabling differential catalysis of hydrolysis and dehydration (Vedernikovs 2011, Cai et al. 2014). For instance, obtaining about 80% of the theoretical maximum of furfural yield from birch wood has been reported. Moreover, most of the cellulose fraction remained intact (Vedernikovs et al. 2010). A commercial-scale furfural process based on this method has been established in 2006 in Iran (Cai et al. 2014). Such a process may enable subsequent production of biofuels, such as ethanol or microbial biodiesel from the solid residue of furfural production. Combined production of furfural and ethanol has been calculated to be very advantageous in terms of fossil oil replacement and greenhouse gas emission (Raman and Gnansounou 2015). However, there are only few studies investigating biofuel production from the remainder of furfural extraction (Cai et al. 2014, Yoo et al. 2012).

In the present study, we investigated the potential of wheat straw after furfural extraction for the co-production of ethanol or biodiesel. After degradation of the residue by standard cellulosic enzymes, the use of the hydrolysate as a substrate for either ethanol- or biolipid production was tested. For the biolipid production, we tested the production potential of two different oleaginous yeasts, the ascomycete *Lipomyces starkeyi* and the basidiomycete *Rhodotorula babjevae*, as there is a huge diversity among the oleaginous yeasts, and optimal yeast strains for each process still need to be established.

## Materials and methods

### Wheat straw hydrolysate

Winter wheat “Zentos” straw was obtained from a local farm close to Riga, Latvia. Its composition and that of the solid remainder after furfural extraction was determined using standard methods (Technical Association of the Pulp, Paper and Converting Industry- TAPPI): extractives (TAPPI 204), hemicelluloses (TAPPI 203 cm-99), lignin (TAPPI 222), and ashes (European Standard EN 14775) (Obolenskaya et al. 1965, Zakis 2008). Cellulose was determined by the Kürschner and Hoffer method (Kürschner and Hoffner 1931). Approximately 2 g of sample was treated with 100 ml of a 1:4 (*v*/*v* nitric acide-ethanol) mixture and then raised to boiling for 1 h. After filtration, the insoluble residue was retreated twice using the same process. Finally, the solid was washed with distilled water until neutrality of the sewage liquid (pH paper coloration). Then the solid was dried to constant weight at 105 ± 3 °C and weighed.

From the wheat straw, furfural was extracted at the pilot plant at the Latvian State Institute of Wood Chemistry. Briefly, the straw was crushed with a Wiley-type mill (Cutting mill SM 100, Retsch GmbH & Co, Haan, Germany), to a particle size 10–20 mm and particles that could pass through a 2-mm sieve and then mixed with an acid catalyst solution (0.1 g catalyst solution per g dry weight of straw were added) using a blade mixer. The obtained material was treated with a stream of water steam during 60 min at 150 °C, in a thermochemical processing unit. Furfural was obtained from the steam condensate (Vedernikovs et al. 2010); the obtained yield was 0.11 g furfural per g dry mass of wheat straw, which is equal to an extraction efficiency of 69% of the maximally possible furfural yield from this material. After water vapour stream treatment, the solid fraction containing the lignocellulosic leftover was washed with distilled water in the same reactor at 110 °C and at a pressure of 0.05 MPa. After a 10-min incubation, the liquid was removed by filtering through a sieve (mesh size 1.5 mm) in the bottom of the reactor. Washing of the residue was repeated four times; the total amount of water was 12 l per kg of solid matter. Subsequently, the material (moisture content 81–83%) was emptied from the reactor and dried at room temperature to a moisture content of 7–10%.

### Enzymatic hydrolysis of the solid residue after furfural extraction

The solid residue from furfural extraction was treated with biomass degrading enzymes to obtain soluble sugar for ethanol and lipid generation. Hydrolysis was performed in a bioreactor as described earlier (Frankó et al. 2015) with some modifications: Treatment was performed with 15 filter paper units Cellic CTec3 enzyme cocktail (Novozyme A/S, Bagsværd, Denmark) per g dry weight at pH 4.8.

### Yeast cultivation

For the different fermentation experiments, the yeast strains *S. cerevisiae* J672 (industrial isolate from Agroetanol AB, Norrköping Sweden, Blomqvist et al. 2010), *R. babjevae* DBVPG 8058 (Industrial Yeasts Collection, Perugia, Italy, originally strain number J195, strain collection of the Department of Microbiology, Swedish University of Agricultural Sciences, isolated from apple), and *L. starkeyi* CBS 1807 were used. The strains were stored in glycerol stocks containing 50% YPD and 50% glycerol at − 80 °C and pre-cultivated on YPD agar plates (20 g/l glucose, 20 g/l peptone, 10 g/l yeast extract, 15 g/l agar) at 25 °C.

For toxicity tests of the enzymatic hydrolysate and to determine the impact of addition of different levels of nitrogen, cells were cultivated at 25 °C on an orbital shaker at 125 rpm in 100 ml Erlenmeyer flasks in three different media: (i) 10 ml of hydrolysate (pure hydrolysate), (ii) 9 mL of hydrolysate plus 1 mL YNB/yeast extract (YNB: yeast nitrogen base without amino acids and ammonium sulphate, Difco™, Becton Dickinson & Co., Stockholm), final concentrations in the medium YNB 2 g/l, yeast extract, 0.75 g/l, ammonium sulphate (Merck, Solna, Sweden) 2 g/L; low-nitrogen variant, or (iii) 9 mL of hydrolysate and 1 mL peptone (Merck)/yeast extract (final concentrations: peptone 20 g/L, yeast extract 10 g/L); high-nitrogen variant. The pH was adjusted to 5.0 using 1 M NaOH. Every 24 h, samples were taken for determining optical density at 600 nm (OD_600_) (Biochrom WPA CO8000 Cell density meter), pH, and the concentrations of sugar and ethanol.

Fermentation experiments for testing the ethanol potential were performed in triplicates, for testing the lipid production, potential fermentations were performed in duplicates, in 0.7-L bioreactors (Multifors, Bottmingen, Switzerland) in 300-ml hydrolysate at 30 °C. To determine the ethanol production potential of the hydrolysate, fermentations were run with *S. cerevisiae*, at an oxygen tension of 0%. The fermenter was flushed with N_2_ before inoculation; during fermentation, the gas flow was switched off. For lipid production, oxygen tension was adjusted to 20%, and fermentations were performed with either *L. starkeyi* or *R. babjevae*. *S. cerevisiae* was inoculated to an initial OD_600_ of 1, both oleaginous yeasts to an OD_600_ of 10. To obtain the inoculum, the yeasts were pre-grown in liquid YPD at 25 °C and 130 rpm on an orbital shaker in baffled 500-ml shake flasks containing 100-ml media. *S. cerevisiae* was pre-cultivated for 24 h. *L. starkeyi* was grown for 72 h and *R. babjevae* for 48 h; before inoculation, the cells were washed twice with saline (9 g/l NaCl solution). The oleaginous yeasts were adapted to nitrogen limitation in YNB media (70 g/L glucose, 1.7 g/L YNB, 2 g/L ammonium sulphate, 0.75 g/L yeast extract) over night at the same conditions as described above.

### Analytical methods

Yeast cell biomass was quantified by OD measurements or direct in 2-mL samples by drying as described in Brandenburg et al. (2016). Correlations were established for OD and dry matter. For *S. cerevisiae*, 1 OD corresponded to a dry matter of 0.37 g, as described earlier (Blomqvist et al. 2010). For *L. starkeyi*, 1 OD corresponded to a dry matter of 0.19 g; for *R. babjevae*, 1 OD corresponded to 0.16 g. The cells were harvested by centrifugation at 17,000 g, washed twice with distilled water, and dried at 105 °C for 48 h. Glucose, acetic acid, glycerol, and ethanol were determined by HPLC as previously described (Fredlund et al. 2004). For a rapid testing, glucose concentration of the medium was measured using Medi-Test Glucose strips (Macherey-Nagel, Düren, Germany) as described earlier (Blomqvist et al. 2012). Lipids of the oleaginous yeasts were extracted using a modified Bligh & Dyer method (Brandenburg et al. 2016, Folch et al. 1957). One hundred milligrams of freeze-dried yeast cells were suspended in 1 ml 1 M HCl, soaked for 15 min and incubated for 1 h at 75 °C. Five milliliters of chloroform:methanol (2:1, *v*/*v*) was added, and the tube was vortexed for 15 min at highest speed. The solution was centrifuged at 5000 g for 3 min at room temperature, and the lipid layer was transferred to a pre-weighted tube. The remaining content of the sample was re-extracted by adding 2.5 ml chloroform, mixing by 1min-vortexing and centrifugation at 3000 g for 5 min. The lipid phase was added to the first extraction, and the solvent was evaporated under N_2_. Lipids were quantified gravimetrically. As lipid extraction is laborious and requires a substantial amount of fermentation medium and because it was not the purpose of this study to follow the kinetics of lipid formation, lipid concentration was only determined at the beginning and end of each fermentation with the oleaginous yeasts. For determination of the fatty acid composition, the fatty acids were methylated by addition of BF_3_ according to Appelqvist (1968), and the methyl esters were analysed by gas chromatography (GC) as recently described (Olstorpe et al. 2014, Brandenburg et al. 2016). Fatty acids were identified according to standard mixture 68A (Nu-Check, Elysian, MN) and retention times. The double binding or unsaturation index (UI) was calculated as UI[%] = [%16:1 + %17:1 + %18:1 + (%18:2)·2 + (%18:3)·3]/100.

Nitrogen in the hydrolysate was quantified by Kjeldahl analysis, using Cu as catalyst (Kjeldahl 1883). Analysis was performed at Swedish University of Agricultural Sciences, Department of Animal Nutrition and Management. Standard deviation of the analysis was stated as maximum 0.9% of the totally determined concentration.

## Results

### Composition of wheat straw and the residue after furfural extraction

The wheat straw used in the experiments contained (see Materials and Methods) 29.4% (± 0.3) hemicellulose, 47.2% (± 0.4) cellulose, and 17.8% (± 0.6) lignin. After furfural extraction (resulting in 110 g furfural per kg total straw dry matter), a solid residue was obtained, corresponding to 67.6% of the wheat straw dry matter.

Analysis of the solid residue identified that it mainly consisted of cellulose (69.9%) of which 4% was decomposed during the furfural extraction process, and lignin (26.3%).

### Enzymatic digest of the solid residue of furfural extraction

Enzymatic degradation of the solid residue was performed, with a final concentration of water insoluble solids (WIS) of 145 g/L. The final glucose concentration from enzymatic hydrolysis was 44.6 g/L, corresponding to 44% of the cellulose present in the solid residue. There were almost no other compounds found in the hydrolysate; no xylose was found, and acetic acid concentration was below 0.1 g/L. The nitrogen content was 82 mg N/L.

### Growth of yeasts on the enzymatic hydrolysate

To check the hydrolysate for inhibitory characteristics, and to check whether an addition of nitrogen is required in the subsequent fermentation steps, the ethanol production yeast *S. cerevisiae* and the oleaginous yeasts *L. starkeyi* and *R. babjevae* were tested for growth in the hydrolysate at different nitrogen additions. All tested strains were able to grow in undiluted hydrolysate. *S. cerevisiae* consumed all sugar during the first 24 h of cultivation under all three different nitrogen supplies. However, there were differences in the final optical density. In pure hydrolysate, *S. cerevisiae* reached a maximum OD_600_ of 12 (± 0.3), at low-nitrogen addition 20 (± 0), and at high-nitrogen addition 40 (± 0). The oleaginous yeasts consumed the glucose slower than *S. cerevisiae*. *L. starkeyi* had consumed all sugar after 48 h in the two cultivations with nitrogen addition and after 72 h in the pure hydrolysate. *R. babjevae* consumed all sugar after 48 h in the cultivation with high nitrogen addition; in hydrolysate with low nitrogen addition and in pure hydrolysate, complete sugar consumption was monitored after 72 h. The maximum obtained OD_600_ was for *L. starkeyi* 17.3 (± 1.1), 20 (± 0), and 40 (± 0) in pure hydrolysate, and hydrolysate with low- and high-nitrogen addition, respectively. *R. babjevae* reached 15.7 (± 1.8), 20 (± 0), and 60 (± 0), respectively.

### Ethanol production from the hydrolysate

**Fig. 1:**
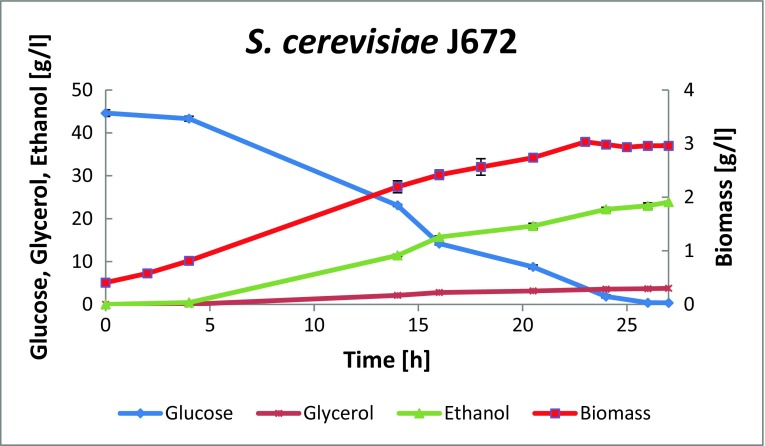
Growth and ethanol production kinetics obtained from pretreated remainder after furfural extraction. Three independent fermentation experiments were performed; mean values and standard deviations are presented

Ethanol production from undiluted hydrolysate was tested in a bioreactor under low-oxygen conditions. The medium was first sparged with N_2_, and then *S. cerevisiae* was added to the fermentation broth to an initial OD of 1.0. The fermentation was followed until the glucose was finished, which happened within 27 h (Fig. 1). The ethanol yield was calculated from the final ethanol concentration divided by the consumed glucose (100% of the glucose was consumed and no ethanol assimilation was observed). In the fermentations, average final ethanol concentrations of 23.85 g/L were obtained, corresponding to a yield of 0.53 g ethanol per g released glucose (± 0.01). This is above the theoretical maximum of the ethanol yield from glucose. Probably, some oligosaccharide residues were still present in the fermentation broth, together with some cellulase enzyme, which released additional sugar monomers during fermentation.

### Lipid production from the hydrolysate

**Fig. 2:**
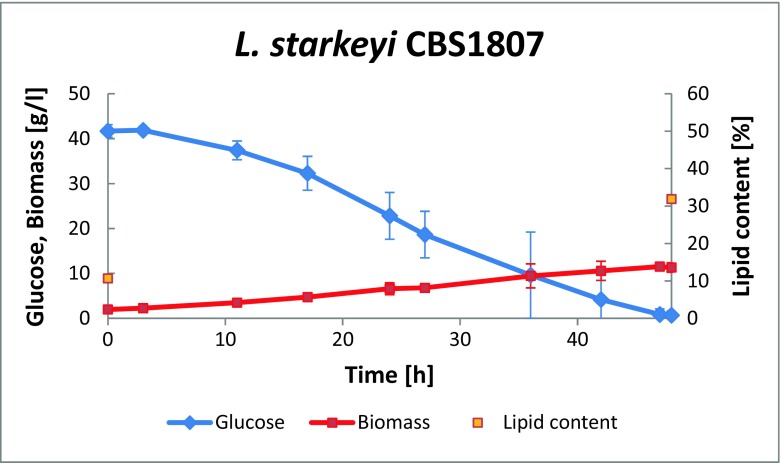
Growth and lipid production kinetics obtained from pretreated remainder after furfural extraction, when using *L. starkeyi* CBS1807. Two independent cultivations were performed; the figure shows mean values and deviation from the mean value

Lipid production was tested using two oleaginous yeast species, the ascomycete *L. starkeyi* and the basidiomycete *R. babjevae*. Both yeasts were after pre-cultivation pre-grown in low-nitrogen medium, to induce the metabolic pathways resulting in lipid accumulation. Lipid formation was calculated from the difference between the amounts of lipids in the yeast cell biomass at the start of the cultivation and after the total consumption of glucose. Cultures were run in duplicates, in undiluted enzymatic hydrolysate. The cultures were inoculated to a dry matter content of 1.95 g/L. Initial lipid content of the cells of *L. starkeyi* was 10.7% of dry matter, corresponding to an initial lipid concentration in the cultures of 0.21 g/L. Glucose was consumed within 48 h; dry matter increased to 11.72 ± 0.46 g/L with a lipid content of 31.89 ± 0.6% (Fig. 2). Thus, a lipid amount of 3.48 ± 0.18 g/L was produced. This corresponds to a total production rate of 0.08 ± 0.01 g/L h and a lipid yield of 0.09 ± 0.01 g per g consumed glucose. The final production of lipid per solid residue was thus 24 g per kg solid residue.

**Fig. 3:**
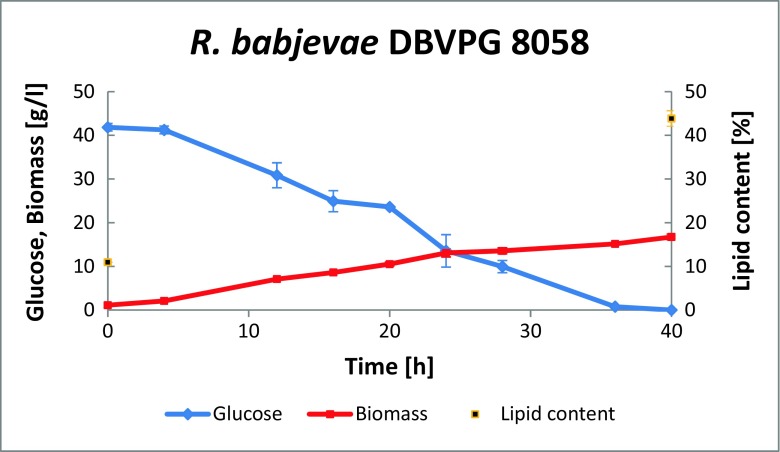
Growth and lipid production kinetics obtained from pretreated remainder after furfural extraction, when using *R. babjevae* DBVPG 8058. Two independent cultivations were performed; the figure shows mean values and deviation from the mean value

*R. babjevae* consumed the glucose already within 40 h. The initial biomass in the parallels was 1.1 g/L, with a lipid content of 10.95%. Thus, the initial lipid concentration in the culture was 0.12 g/L. After 40 h, biomass dry matter was 16.48 ± 0.4 g/L, with a lipid content of 43.9 ± 1.32% (Fig. 3). This corresponded to an amount of produced lipids of 7.14 ± 0.02 g/L, a lipid production rate of 0.18 ± 0.0 g/h, and a lipid yield of 0.17 ± 0.0 g per g consumed glucose. Thus, when using *R. babjevae* to convert the hydrolysate to lipids, 49.2 g lipids per kg solid residue were obtained.

### Fatty acid profiles of *L. starkeyi* and *R. babjevae*

The fatty acid profiles of the two oleaginous yeasts at the start and the end of the fermentations are shown in Table 1. Both yeasts showed substantial differences in their overall fatty acid composition. At the beginning of the cultivation, the major fatty acid was oleic acid (C18:1) in both yeast, but in *R. babjevae*, this fatty acid contributed more than 60.0% of all fatty acids, while it was 44.1% in *L. starkeyi*. Other substantial fatty acids in *L. starkeyi* at the beginning of fermentation were palmitic acid (C16:0, 32%), palmitoleic acid (C16:1, 5.2%), stearic acid (C18:0, 6.8%), and linoleic acid (C18:2, 9.2%). After cultivation of *L. starkeyi* in the hydrolysate, a variety of changes in the fatty acid profile were observed. There was a slight decrease in the number of saturated and monounsaturated fatty acids, while the proportion of linoleic acid increased to 18.9%. Moreover, a detectable amount of α-linolenic acid (C18:3 n-3, 2.6%) was observed.

**Table 1:** Fatty acid profile and unsaturation index (UI) of *Lipomyces starkeyi* and *Rhodotorula babjevae* during growth on hydrolysate at the beginning and the end of the fermentation (fermentation was ended when the glucose was depleted)

Species, incubation time [h]	Fatty acids, proportion [%] of the total amount of fatty acids (numbers in parentheses represent standard deviations)								UI
	C16:0	C16:1	C17:1	C18:0	C18:1	C18:2	C18:3 (n-6)	C18:3 (n-3)	
*L. starkeyi*, 0	32.0 (± 1.2)	5.2 (± 1.7)	1.5 (± 0.7)	6.8 (± 3.5)	44.1 (± 2.3)	9.2 (± 4.4)	b.d.^a^	b.d.^a^	0.69
*L. starkeyi*, 48	27.0 (± 2.1)	3.7 (± 0.9)	0.9 (± 0.1)	4.0 (± 1.9)	40.9 (± 0.8)	18.9 (± 3.8)	b.d.^a^	2.6 (± 0.7)	0.91
*R. babjevae*, 0	16.0 (± 0.6)	1.3 (± 0.0)	0.3 (± 0.2)	4.5 (± 0.2)	63.4 (± 0.4)	7.1 (± 0.3)	b.d.^a^	2.6 (± 0.7)	0.87
*R. babjevae*, 40	10.3 (± 0.7)	0.7 (± 0.1)	21.8 (± 1.5)	1.6 (± 0.1)	23.3 (± 0.9)	17.0 (± 0.7)	13.3 (± 1.1)	5.0 (± 0.3)	1.35

^a^Below detection limit

In *R. babjevae*, at the beginning of the cultivation apart from the dominating oleic acid (63.4%), palmitic acid (16.0%), stearic acid (4.5%), and linoleic acid (7.1%) were found in noteworthy proportions. After a 40-h cultivation, the proportion of oleic acid had substantially decreased and was with 23.3% in the range of heptadecenoic acid (C17:1, 21.8%). C17:1 was the fatty acid with the largest increase, as it was only present with a very low proportion (1.5%) at the beginning of the cultivation. Also in the basidiomycetous yeast, there was a tendency that the proportion of saturated fatty acids and most of the single unsaturated (except C17:1) decreased. Polyunsaturated fatty acids increased, including linoleic acid, α- and γ-linolenic acid, and the latter up to a proportion of more than 10% (Table 1).

## Discussion

Current industrial methods for furfural production tend to damage the cellulose fraction of the residual material, which makes it impossible to produce biofuels from these residues (Cai et al. 2014, Machado et al. 2016). In this study, we investigated the biofuel potential of the cellulosic residue after furfural extraction, using the method developed at the Latvian Institute of Wood Chemistry (Vedernikovs 2011), where 110 g furfural have been extracted per kg wheat straw. Enzymatic treatment hydrolysed 44% of total cellulose. This can be assumed as a good proportion, compared to other studies on wheat straw. From wheat straw pretreated with diluted acid, only 25% of the cellulose could be degraded to glucose (Pedersen et al. 2011). In experiments of integrated storage and microbial pretreatment, 25–50% of the cellulose was converted to glucose (Passoth et al. 2013). Probably, even higher values of cellulose degradation might be possible when using simultaneous saccharification and fermentation, to avoid feedback inhibition of cellulase, and in general, by using improved enzyme cocktails (Atreya et al. 2016).

The enzymatic hydrolysate had almost no inhibitory potential on the yeasts used in this study. Obviously, the furfural extraction and the washing step at the end of the thermochemical treatment had removed most fermentation inhibitors. Accordingly, all three yeasts, including the two more inhibitor sensitive oleaginous species could grow on undiluted hydrolysate. In earlier experiments, hydrolysate had to be diluted by a factor of 3–10, to get non-adapted yeasts to grow (Blomqvist et al. 2011, Tiukova et al. 2014, Brandenburg et al. 2016). In the ethanol fermentation, the theoretical maximum of ethanol production was reached; in fact, slightly more ethanol was produced than it could be expected according to the measured amount of glucose in the medium. This was probably due to some cellulase activity present in the hydrolysate, and due to this, sugar releases during fermentation.

Our study, apart from testing the ethanol potential, represents the first attempt to generate lipids from the solid residue of furfural production. We found considerable differences between the two tested yeast strains when grown on the tested substrate, with *R. babjevae* (lipid yield 0.17 and production rate 0.18 g/L h) being superior over *L. starkeyi* (0.09 and 0.08 g/L h). The physiological basis of this difference on the same substrate is not clear; however, it fits well to results on other lignocellulose hydrolysate, where *L. starkeyi*-strains produced rather low lipid amounts compared to other oleaginous yeasts (Slininger et al. 2016). The order of magnitude of the lipid yield of *L. starkeyi* was similar to a recent study in our group, where we obtained the highest lipid yield from hemicellulose hydrolysate that has been reported so far (Brandenburg et al. 2016). Between the two tested species, *R. babjevae* seems to be the yeast of choice in terms of further research for biofuel production from lignocellulose. The obtained lipid yield- and -production rate were also relatively high compared to other oleaginous yeasts, cultivated on either lignocellulosic hydrolysate or a glucose-based model substrate (Slininger et al. 2016, Shen et al. 2013). In recent system analyses, we pointed out that rapid conversion of the substrate to lipids has great impact on the sustainability of the conversion, because aerobic cultivations, as required for lipid production, require much energy, implying that a faster fermentation will largely improve process efficiency (Karlsson et al. 2016, Karlsson et al. 2017).

The fatty acid profiles changed during the cultivation; especially an increase of the unsaturation index (UI)- reflecting the desaturation of fatty acids was observed. Previous studies have shown an increase of the UI as a response to lowered cultivation temperatures, which is keeping the membrane fluidity on an according level (e.g. Beltran et al. 2008, Rossi et al. 2009, Amaretti et al. 2010, Olstorpe et al. 2014). However, in our experiments, the cultivation temperature was 30 °C, and thus low temperature cannot be the reason for increased desaturation. Moreover, in oleaginous yeasts, membrane lipids represent only a small proportion of the total lipid content; most of the lipids must thus have been storage lipids. It is unclear why the proportion of unsaturated fatty acids increased in both yeasts, but since both yeasts belong to different fungal phyla and have a very high phylogenetic distance, this increase seems to be a conserved response. There was also a remarkable increase of heptadecenoic acid, C17:1 in *R. babjevae* during growth on hydrolysate. The occurrence of this fatty acid in yeasts has rarely been reported (Beltran et al. 2008, Rossi et al. 2009, Tronchoni et al. 2012); however, we have recently found considerable amounts in the ascomycetes *Blastobotrys adeninivorans* and *Wickerhamomyces anomalus* (Olstorpe et al. 2014). In the present study, we found detectable amounts of this fatty acid which were present in both *L. starkeyi* and *R. babjevae*. The background and physiological importance of the strong increase of C17:1 in *R. babjevae* are not clear. Odd numbered fatty acids are produced by using propionyl-CoA in the initial step of fatty acid synthesis (Tehlivets et al. 2007). Very recently, it has been reported that the synthesis of C17:1 increased in yeasts, including in the close relative to *R. babjevae*, *Rhodotorula glutinis*, when carbon sources with odd numbers of carbon atoms were provided, compared to those with even numbers. There was also a slight increase during growth on acetic acid (Rezanka et al. 2015). However, in our experiments, the concentration of carbon sources with odd numbers of carbon atoms should be rather low, and also the amount of acetic acid was much lower than that of glucose. Nevertheless, the increase in C17:1 might open another interesting application of *R. babjevae*, since this fatty acid may have a variety of applications in pharmacy or as antimicrobial agent (Rezanka et al. 2015).

In both oleaginous yeasts studied, the linolenic acid (C18:3) content increased when grown on hydrolysate. *L. starkeyi* formed only C18:3 n-3 (α-linolenic acid), while *R. babjevae* formed both C18:3 n-3 and – n-6 (γ-linolenic acid). Formation of linolenic acid (not specified whether α- or γ-linolenic acid) has been observed in *L. starkeyi* as a response to low cultivation temperatures (Suutari et al. 1996). In *R. glutinis*, highest production of linolenic acid has been observed under non-nitrogen limiting conditions (i.e. under conditions where no lipid was accumulated) or at lowered growth temperatures (25 vs. 30 °C) (Granger et al. 1992). Our experiments were running at 30 °C, and the fact that the lipid content of the cells more than doubled during the cultivation argues against a missing nitrogen limitation. Production of C18:3 up to 5% of total fatty acids has been reported for *R. babjevae* grown on glycerol (Munch et al. 2015). Production of both α- and γ-linolenic acid was much higher in strain *R. babjevae* DBVPG 8058 grown on the residue of furfural production from wheat straw. When producing biodiesel, a high degree of saturation is desirable, except when the diesel is made for utilisation in low temperatures (Knothe 2005). If the lipids are produced as food or feed additives, polyunsaturated fatty acids such as α-linolenic acid are often regarded advantageous (Das 2006). Further investigation is required to understand the mechanisms regulating desaturation in oleaginous yeasts when growing on lignocellulose hydrolysates.

Only few reports about lipid production in yeasts differentiate between α- and γ-linolenic acid. Granger et al. (1992) only reported α- linolenic acid, (Munch et al. 2015) did not differentiate between α- and γ-linolenic acid production in *R. babjevae*. α- and γ-linolenic acid have been observed in *W. anomalus* and *B. adeninivorans* (Olstorpe et al. 2014), which are both not known as oleaginous yeasts. The mechanisms behind formation of α- and γ-linolenic acid need further investigation. In experiments with strain *R. babjevae* DBVPG 8058 and other oleaginous yeasts, we found that the ability of *R. babjevae* DBVPG 8058 to form γ-linolenic acid was quite unique for this strain (unpublished results). γ-linolenic acid is used as dietary food supplement and might influence inflammatory responses (Sergeant et al. 2016), and thus, the ability of this strain to produce γ-linolenic acid is of interest for potential biotechnological applications.

This study demonstrates that the method of furfural extraction developed at the Latvian State Institute of Wood Chemistry indeed allows coproduction of furfural and ethanol or lipids from wheat straw. Combined production of furfural and ethanol has been calculated to be very advantageous in terms of fossil oil replacement and greenhouse gas emission (Raman and Gnansounou 2015). Out of 1 kg of wheat straw, apart from 110 g of furfural, 111 g of ethanol or 33 g of lipids (when using *R. babjevae*) could be produced. These additional products can add value to the whole process, and either be used as biofuels or as source of valuable chemicals, for instance, heptadecenoic acid, α- and γ-linolenic acid and by these make the production of the platform chemical furfural, which is solely derived from renewable, lignocellulosic matter, economically more attractive. Utilisation of the remainder of the process as substrate for biogas production, fertiliser, or as burning material to obtain process energy may further increase the efficiency of the process and reduce greenhouse gas emission and dependency on fossil energy (Karlsson et al. 2016, Karlsson et al. 2017).
